# Correlations between ADL in patients with SCI and caregiver burden, quality of life, and presenteeism in South Korea

**DOI:** 10.1038/s41598-023-50559-6

**Published:** 2024-02-07

**Authors:** Sung Shin Kim, On Yoo Kim, Sun Hong Kim, Jae Eun Heo, Seung Hee Ho, Ju Hee Kim, Young-Hyeon Bae

**Affiliations:** 1 Department of Clinical Rehabilitation Research, National Rehabilitation Center, Seoul, Republic of Korea; 2 Department of Rehabilitation Medicine, National Rehabilitation Center, Seoul, Republic of Korea; 3 Department of Nursing, National Rehabilitation Center, Seoul, Republic of Korea; 4 Department of Rehabilitative and Assistive Technology, National Rehabilitation Center, Seoul, Republic of Korea; 5 Department of Healthcare and Public Health, National Rehabilitation Center, Seoul, Republic of Korea

**Keywords:** Public health, Quality of life, Health occupations

## Abstract

The correlations between activities of daily living (ADL) among patients with spinal cord injury (SCI) and their caregivers’ burden, quality of life (QoL), and presenteeism was investigated. Participants included outpatients and inpatients with SCI at a rehabilitation center and their caregivers, recruited between March 2020 and April 2021. Eighty-seven valid responses were analysed using independent *t*-tests and Pearson’s correlations. There was a difference in caregiver burden according to patients’ ADL performance. QoL was negatively correlated with caregiver burden and presenteeism. Caregiver burden and presenteeism were positively correlated. Social support can improve caregivers’ QoL and reduce caregiver burden and presenteeism-induced work impairment.

## Introduction

Spinal cord injury (SCI) is characterized by damage to the motor, sensory, and autonomic nerves. Depending on the extent of the impairment, individuals with SCI experience difficulties walking independently and performing activities of daily living (ADL)^1^. As caregivers of individuals with SCI devote much time to assisting these individuals with ADL, they often have very little time for themselves^2^. Thus, caregivers need sufficient energy to help individuals with SCI maintain healthy lives in the community^3^. Further, caregivers of patients with SCI may experience the same levels of physical, psychological, and emotional stress as the patients^4–6^.

Caregiver burden is defined as a range of stressors that impact emotional, social, financial, physical, and spiritual functioning^7–9^. Caregivers of patients with SCI are frequently exposed to physical burden by being engaged in the care activities of assisting patients; thus, caregiver burden negatively affects caregivers’ health status^10,11^. Such health deterioration can negatively affect caregivers’ physical, mental, and social quality of life (QoL)^10,12,13^. In addition, prolonged caregiving for patients with SCI can lower caregivers’ QoL^14^.

When caregivers’ QoL remains poor, they can develop health issues^15^. While older caregivers experience more difficulties providing care for patients owing to their own health issues and physical limitations, they usually cannot give up their caregiver role^16^. Continuing to perform a job despite such physical or mental strain is referred to as presenteeism^17^. Over time, presenteeism leads to a decline in health and, consequently, absences from work^18^.

Nevertheless, few studies have examined the relationship between ADL performance in patients with SCI and caregiver burden, QoL, and presentism. Therefore, the purpose of this study was to investigate the 1) differences in caregiver burden, QoL, and presenteeism by patients’ ADL and 2) correlations between presenteeism, QoL, caregiver burden, and ADL.

## Methods

### Participants

Participants were recruited in dyads between March 2020 and April 2021 from inpatients and outpatients with SCI at a Korea National Rehabilitation Center and their caregivers. The sample size was determined using G*Power 3.1 software. For an F-test multiple regression analysis with a medium effect size, significance level of 0.05, power of 0.80, and four predictors, the minimum necessary sample size was 85. Thus, the sample size for this study was set at 90 pairs, considering a potential dropout rate of 5%.

Participant selection criteria included a caregiver who provided 1:1 care to patients with SCI, with comprehension and communication skills in the Korean language, and patients with SCI aged 19–79 years. Among individuals who met these three criteria, those who agreed to participate were selected. Exclusion criteria were patients with conditions other than SCI, caregivers who provided care to multiple patients, individuals with cognitive impairment or comprehension problems that could inhibit survey completion, caregivers who were younger than 19 years or older than 79 years, and those who refused to participate. The questionnaire survey was conducted among 47 inpatient–caregiver and 43 outpatient–caregiver pairs. After eliminating two pairs who withdrew and one pair who did not complete the survey, 87 dyads were included in the analysis: 46 inpatient–caregiver and 41 outpatient–caregiver pairs. All participants provided written informed consent. And the study protocols strictly adhered to the Declaration of Helsinki for human subject involvement. Also, all methods were performed in accordance with relevant guidelines and regulations.

### Data collection

Prior to data collection, written consent for voluntary participation was obtained from the caregivers and patients from March 2020 to April 2021.

We used a structured questionnaire comprising questions about the ADL of patients with SCI and caregivers’ general characteristics, caregiver burden, QoL, and presenteeism. The survey took 40–60 min to complete. The participants were informed that the questionnaire required personal information and that they could withdraw from the survey at any time without any disadvantages. To prevent response bias from the participants, one researcher read the questions aloud. The patients and their caregivers completed the survey in separate rooms.

### Instruments

#### Caregiver burden

Caregiver burden was measured using the Caregiver Burden Inventory^19^. The questionnaire consists of five subcategories: time-dependence burden (five items), developmental burden (four items), physical burden (four items), social burden (five items), and emotional burden (five items). Each item is rated on a five-point Likert scale (minimum 1 point, maximum 5 points). The scores range from 25 to 125 points, with higher scores indicating a higher level of caregiver burden. Cronbach’s α in this study was 0.947.

#### ADL

ADL performance was measured using the Modified Barthel Index (MBI)^20^. The tool consists of 11 items rated from 0 to 5. Total scores range from 0 to 100, with 0 indicating total dependency and 100 indicating complete independence in ADL performance. An ADL score of 24 or lower corresponds to the first level of disability (highest dependence) in the disability rating criteria of the 2010 Korean Welfare for Persons with Disabilities Act enforcement regulations. Reliability of the MBI, indicated by Cronbach’s alpha, was 0.944 in this study.

#### World Health Organization-QoL assessment

The Korean version of the Quality-of-Life Assessment from the World Health Organization (Geneva) comprises 26 facets across six domains: 24 facets focus on physical health (three facets), psychological health (five facets), level of independence (four facets), social relationship (three facets), environment (eight facets), and spirituality/religion/personal beliefs (one facet), while two facets are related to general health and overall QoL. Cronbach’s α was 0.859 in a previous study^21^ and 0.901 in this study.

#### Presenteeism

Presenteeism was measured using 10 Likert-type questions from the 13-items Stanford Presenteeism Scale^22^. Responses were rated on a five-point scale ranging from 1 (always) to 5 (never). A higher score indicates a higher level of work impairment. Items 2, 5, 6, 8, and 10 are reverse-coded. For analysis, this study used a 100-point conversion method, (total score − 10)/40 × 100, as recommended by the developer of the original tool. Cronbach’s α was 0.837 in a previous study^21^ and 0.848 in this study.

### Statistical analysis

Frequencies (%), means, and standard deviations of the following variables were calculated: ADL performance of patients with SCI and caregivers’ general characteristics, caregiver burden, QoL, and presenteeism. To examine differences in caregiver burden based on the ADL performance of patients with SCI, independent *t*-tests were performed. To examine differences in caregiver burden based on the level of injury (cervical, thoracic, and lumbar), American Spinal Injury Association Impairment Scale (AIS; A, B, C, D), severity of injury (complete/incomplete, tetraplegia/paraplegia), and post injury period of patients with SCI (3 year less, 3 to 10 years, over 10 years), ANOVA were performed. To examine correlations between the ADL performance of patients with SCI and caregivers’ general characteristics, caregiver burden, QoL, and presenteeism, Pearson’s correlation analysis was conducted. SPSS version 19.0 (IBM Corp., Armonk, NY, USA) was used for data analysis. The significance level was set at *p* < 0.05.

### Ethics declarations

The Institutional Review Board at the National Rehabilitation Center approved this study (NRC-2020-02-012). All study participants had been invited to participate in the study and informed that participation is on their own choice and that they were free to withdraw at any time.

## Results

### Characteristics of individuals with SCI and caregivers

The sex, age, body mass index (BMI), level of injury, AIS grade (A to D), severity of injury, MBI score, and post-injury period of people with spinal cord injury were described in Table 1. The mean (standard deviation) of ADL score for patients with SCI was 35.49 (± 31.43). The general characteristics and related variables for caregivers were shown in Table 2. The mean (standard deviation) of caregiver burden were 64.97 (± 22.72).

**Table 1:** Characteristics of individuals with SCI (*N* = 87).

Category	Variable	Categories	*n* (%)
Individuals with spinal cord injury	Sex	Male	60 (69.0)
		Female	27 (31.0)
	Age (years)	< 50	60 (69.0)
		≥ 50	27 (31.0)
		M ± SD	51.70 ± 17.35
	BMI (kg/m^2^)	< 18.5	7 (8.0)
		18.5–22.9	42 (48.3)
		> 23.0	38 (43.7)
		M ± SD	22.82 ± 3.33
	ADL score (MBI)	≤ 24	45 (51.7)
		25–49	10 (11.5)
		50–74	19 (21.8)
		≥ 75	13 (14.9)
		M ± SD	35.49 ± 31.43
	AIS grade	AIS A	39 (44.8)
		AIS B	22 (25.3)
		AIS C	11 (12.6)
		AIS D	15 (17.2)

*MBI* modified Barthel index, *ADL* activities of daily living, *AIS* American Spinal Injury Association Impairment Scale.

**Table 2:** General characteristics and related variables for caregivers (N = 87).

Variable	Mean ± SD
Age (years)	57.29 ± 11.18
Period of care (years)	5.74 ± 7.64
Time spent providing care (hours per day)	19.64 ± 7.32
Caregiver burden (scale)	64.97 ± 22.72
QoL (scale)	49.63 ± 8.54
Presenteeism	34.87 ± 13.61

*QoL* quality of life, *SD* standard deviation.

### Caregiver burden according to the characteristics of individuals with SCI

Caregiver burden according to the characteristics of individuals with SCI were shown in Table 3. There are no significant differences in level of injury, AIS grade, severity of injury (complete/incomplete, tetraplegia/paraplegia), and post injury period (Table 3).

**Table 3:** Caregiver burden according to characteristics of individuals with SCI (*N* = 87).

Variable	Categories	*n* (%)	Care burden	*F*	*p*
Level of injury	Cervical	45 (51.7)	66.51 ± 17.29	2.058	.134
	Thoracic	33 (37.9)	59.48 ± 22.94		
	Lumbar	9 (10.3)	54.22 ± 20.86		
Severity of injury (AIS)	AIS A	39 (44.8)	59.87 ± 17.72	.889	.450
	AIS B	22 (25.3)	67.45 ± 26.96		
	AIS C	11 (12.6)	66.36 ± 13.58		
	AIS D	15 (17.2)	59.67 ± 18.87		
Severity of injury (with level of injury)	Complete tetraplegia	29 (33.3)	67.45 ± 19.17	1.250	.297
	Incomplete tetraplegia	16 (18.4)	64.81 ± 13.65		
	Complete paraplegia	32 (36.8)	58.22 ± 23.02		
	Incomplete paraplegia	10 (11.5)	58.80 ± 21.33		
Post injury period (years)	< 3	46 (52.9)	63.24 ± 16.78	0.175	.840
	3–10	13 (14.9)	64.15 ± 27.11		
	> 10	28 (32.2)	60.75 ± 22.35		
	M ± SD	7.51 ± 8.25			

*AIS* American Spinal Injury Association Impairment Scale.

### Comparative analysis of caregiver burden, QoL, and presenteeism with SCI ADL

In the comparative analysis of caregiver burden, QoL, and presenteeism in relation to patients’ ADL scores, those who scored less than 24 points in ADL performance showed a significantly higher caregiver burden than those who scored 24 or more (*p* = 0.01). The results showed no significant differences in either QoL or presenteeism (Table 4).

**Table 4:** Differences in presenteeism, caregiver burden, and QoL according to ADL scores.

	ADL < 24	ADL ≥ 24	*t*	*p-*value
Presenteeism	36.04 (± 12.02)	32.86 (± 16.26)	0.691	0.494
Caregiver burden	72.04 (± 52.86)	52.86 (± 20.42)	2.719	0.010
QoL	49.05 (± 7.97)	50.62 (± 9.67)	0.542	0.591

*ADL* activities of daily living, *QoL* quality of life.

### Correlations between care-related variables (caregiver burden, QoL, presenteeism, and ADL)

An analysis of the correlations between care-related variables showed a highly significant negative correlation between QoL and caregiver burden (coefficient = −0.772, *p* < 0.01) and a moderately significant negative correlation between QoL and presenteeism (coefficient = −0.546, *p* < 0.01). Moreover, there was a moderately significant positive correlation between caregiver burden and presenteeism (coefficient = 0.581, *p* < 0.01; Table 5).

**Table 5:** Relationships among care-related factors.

	Presenteeism	QoL	Caregiver burden	ADL
Presenteeism	1			
QoL	−0.546*	1		
Caregiver burden	0.581*	−0.772*	1	
ADL	−0.107	0.105	−0.377	1

*ADL* activities of daily living, *QoL* quality of life.

*p < 0.01.

## Discussion

Depending on the degree of motor and sensory impairment among patients with SCI, they may experience difficulties in life that make them entirely dependent on caregivers; conversely, they may be able to independently perform most ADL. As a result of these difficulties, patients with SCI also often experience psychological problems, such as anxiety and depression^23,24^. They can become dependent on others owing to physical disabilities, which may lead them to experience negative mental states such as shame, helplessness, low self-esteem, and depression. Owing to decreased contact with others, they gradually become isolated and lose their social roles^25^. The problem appears not only in patients with SCI but also in family caregivers. The families of individuals with SCI who require long-term care experience considerable caregiver burden in terms of time as well as financial, social, physical, and emotional aspects^26^. This study examined the correlation between ADL performance in patients with SCI and their caregivers’ caregiver burden, QoL, and presenteeism in rehabilitation centers and the community. Therefore, this study obtained baseline data to help provide social support to caregivers.

Regarding ADL scores, caregivers of patients who scored 24 or lower showed significantly higher caregiver burden than did caregivers of patients who scored 25 and above. In previous studies, caregiver burden decreased as the physical function of individuals with SCI improved, which supports the current findings^27–29^. This is relevant in that people with ADL scores of 24 or below are completely dependent, need long-term care management, and have difficulties integrating into society. Consequently, such a high level of dependency may affect caregiver burden.

Scholten et al. report an average age of 47 years for caregivers of patients with SCI^30^. In the present study, the average age was higher: 57 years. According to the 2021 SCI data released by the National SCI Statistical Center, the average age of patients with SCI increased from 29 years in the 1970s to 43 years in 2015^31^. A South Korean study also reports that the average age of individuals with SCI has increased^27^. This increase in age may be linked to the increase in the average age of caregivers, who are typically spouses or parents. Caregivers were in their late 50s, and their caregiver burden increased with age. This is similar to the findings of Gajraj-Singh^28^. In addition, among the subcategories of caregiver burden, time dependency was the highest, compared to other types of burden, including physical burden. The burden for time dependency was also high: the average amount of time spent providing care was 19.64 h per day and 5.74 years. Similarly, a study conducted among 163 personal caregivers of individuals with SCI reported that caregiver burden increased as the SCI condition and the caregiving task were prolonged^14^. More than 50% of the participants were sole caregivers who provided around-the-clock care and there was a positive correlation between caregiver burden and caregiving time (the more hours spent on care, the higher the burden)^29^.

There was a highly significant negative correlation between QoL and caregiver burden and a moderately significant negative correlation between QoL and presenteeism. Further, there was a moderately significant and positive correlation between caregiver burden and presenteeism. Thus, caregivers of patients with SCI experience not only serious caregiver burden but also a physical burden—for example, musculoskeletal pain—owing to the constant tasks necessary to provide physical assistance, including changing the patient’s position or moving them to prevent bedsores. As these caregivers provide long-term care for patients, they often experience fatigue and sleep deprivation and develop negative physical and psychological responses, such as depression, owing to the deterioration of their own health^32^. Specifically, caregivers’ health difficulties included fatigue; swelling; and musculoskeletal pain in the shoulders, lower back, neck, and feet. The causes of such health problems were reported to be work circumstances that require the caregivers to constantly work on their feet and move around, as well as caregiving tasks that require them to transfer patients or use their bodies^33^.

In the relationship between caregivers’ health problems and presenteeism, there was a significant positive correlation when a caregiver had more than one health problem. In previous studies, when the number of health problems increased from one to three, a strong positive correlation with presenteeism was observed^34,35^. With one to two health problems, the scale of work impairment was so trivial that workers could not recognize it; however, as the number of health problems increased, the effect became relatively more severe, leading to absenteeism. This supports the current findings. In addition, health problems have a strong correlation with decreased work performance, and caregivers’ health issues affect their job productivity^36^. Finally, based on the correlation between caregivers’ presenteeism and QoL, the findings indicated that caregiver burden, QoL, and presenteeism were closely related. Therefore, it seems necessary to provide social and institutional support to reduce the burden among caregivers of patients with SCI. Additionally, social policies should be developed with a focus on distributing devices and care robots to reduce caregiver burden and provide support to caregivers.

The limitations of this study are that the participants were limited to a single institution in a single region, and the average ADL score of patients with SCI was approximately 35, making it difficult to compare them across a wide range. In future research, if participants at various levels of ADL performance from many institutions are recruited, the findings may be more generalizable.

## Conclusions

After examining the correlations between caregivers’ burden, QoL, and presenteeism and the ADL performance of patients with SCI, significant differences in caregiver burden based on ADL scores (with 24 points as a threshold) were observed. Among the care-related variables, QoL increased as caregiver burden and presenteeism decreased, indicating a correlation between caregiver burden and presenteeism. The findings suggest the need for social support to improve caregivers’ QoL and reduce their burden and presenteeism-induced work impairment.

## Data Availability

The data used and/or analyzed during the current study are available from the corresponding author on request.

## Abbreviations

SCI: Spinal cord injury
ADL: Activities of daily living
QoL: Quality of life
IRB: Institutional Review Board
SD: Standard deviation
